# Selective Nonenzymatic Amperometric Detection of Lactic Acid in Human Sweat Utilizing a Multi-Walled Carbon Nanotube (MWCNT)-Polypyrrole Core-Shell Nanowire

**DOI:** 10.3390/bios10090111

**Published:** 2020-08-28

**Authors:** Young Min Choi, Hana Lim, Ho-Nyun Lee, Young Min Park, Jin-Seong Park, Hyun-Jong Kim

**Affiliations:** 1 Surface Technology Group, Korea Institute of Industrial Technology (KITECH), Incheon 21999, Korea; dudalszha@gmail.com (Y.M.C.); hana0313@kitech.re.kr (H.L.); hnlee@kitech.re.kr (H.-N.L.); 2 Division of Materials Science and Engineering, Hanyang University, Seoul 04763, Korea; jsparklime@hanyang.ac.kr

**Keywords:** polypyrrole, lactic acid, sweat, nonenzymatic, amperometric, selectivity

## Abstract

Lactic acid plays an important role as a biochemical indicator for sports medicine and clinical diagnosis. The detection of lactic acid in sweat is a promising technique without any intrusive inconvenience or risk of infection. In this study, we present a selective nonenzymatic amperometric detection method for lactic acid in human sweat utilizing a multi-wall carbon nanotube (MWCNT)-polypyrrole core-shell nanowire. Because polypyrrole is a p-type conducting polymer, onto which anions are exclusively doped, leading to charge transfer, it offers selective detection for lactate anions at a specific potential, while being inert to the neutral and cationic species contained in human sweat. A chronoamperometric study reveals good sensing performance for lactic acid with a high sensitivity of 2.9 μA mM^−1^ cm^−2^ and detection limit of 51 μM. Furthermore, the MWCNT-polypyrrole nanowire exhibits excellent selectivity for lactic acid over interfering species, such as sodium chloride, glucose, urea, and riboflavin, which coexist with lactic acid in sweat. Finally, a nonenzymatic amperometric sensor for the selective detection of lactic acid in human sweat is demonstrated on commercial flexible electrodes. The results demonstrate the potential applications of the MWCNT-polypyrrole core-shell nanowire as a nonenzymatic amperometric lactate sensor.

## 1. Introduction

Lactic acid is an important metabolite in clinical cases because it indicates the status of metabolic impairment under anaerobic conditions. Energy demands that cannot be met by aerobic respiration induce anaerobic metabolism, consequently increasing the concentration of lactic acid in tissue. Therefore, levels of lactic acid have been widely used for determining athletic training status and fitness in sports medicine. Furthermore, lactic acid plays an important role as a biochemical indicator for various diseases and symptoms, such as sepsis, acute cardiac disorders, and lactate acidosis [1]. Lactic acidosis, which result from abnormal high levels of lactic acid, occurs in the diseases mentioned above. Furthermore, the lactate level could be used as an indicator of the acid-based (pH) balance when monitoring patients. The normal concentration of lactic acid in human blood is in the range of 0.5~2.2 mM at rest, but this value can increase to over 30 mM during intense physical activity as muscle cells switch to anaerobic metabolism [2,3].

To profile lactic acid levels during intense exertion, blood must be collected repeatedly at frequent intervals. This not only generates intrusiveness and inconvenience, but also increases the risk of infection. This is why lactic acid monitoring is not conducted during daily sports training, despite the importance of lactic acid in sports medicine [4]. In addition to its presence in blood, the concentration of lactic acid in human sweat is in the range of 4~25 mM at rest and can increase to over 50 mM during intense physical activity [5]. It is well known that an increase in lactic acid in blood leads to a corresponding increase in lactic acid in sweat, facilitating non-invasive quantification of lactic acid levels [6]. Accordingly, the detection of lactic acid in sweat is an excellent measurement technique without intrusive inconvenience or risk of infection.

For detecting lactic acid, electrochemical approaches have attracted significant attention based on their high sensitivity and selectivity, low detection limit, compatibility with miniaturization, and ease of use. The most commonly used electrochemical lactic acid sensor utilizes immobilized enzymes, such as lactate oxidase (LOx) and lactate dehydrogenase (LDH). The LDH enzyme electrochemically catalyzes redox reactions between lactate and pyruvate in the presence of nicotinamide adenine dinucleotide (NAD+). The reduced form of NAD+ (NADH) can then be detected amperometrically [7,8]. However, enzymatic electrochemical sensors are affected by the influences of various environmental factors, such as temperature, oxygen, pH, humidity, and organic reagents, based on the inherent instability of enzyme molecules, which affects the sensitivity and reproducibility of such sensors [9]. High costs, mandatory low-temperature storage, and complicated fabrication procedures are also significant drawbacks. Although a few studies reported nonenzymatic detection of lactic acid, selective detection has not been demonstrated [6,10].

**Figure 1:**
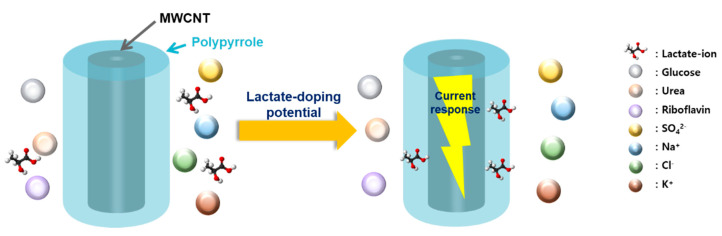
Schematic illustration for selective detection of lactic acid without interference of neutral and cationic species.

In this study, the concentration of lactic acid in sweat was determined electrochemically, utilizing a multi-wall carbon nanotube (MWCNT)-polypyrrole core-shell nanowire. Polypyrrole is a conventional p-type conduction polymer. The p-type conduction polymers are exclusively doped with anions, such as lactate, at specific potentials, based on redox processes and charge transfer phenomena [11]. Consequently, the electrochemical doping of lactate anions onto a p-type conducting polymer at fixed potential is expected to induce a current response that can be utilized to detect lactic acid. Additionally, because neutral and cationic components cannot be doped onto p-type conducting polymers, such polymers are inert to the neutral and cationic species contained in human sweat. These features of p-type conducting polymers make them suitable for the selective amperometric detection of lactic acid without interference from the other metabolites found in sweat, as shown in Figure 1. We utilized polypyrrole as a representative p-type conductive polymer and amperometrically determined the concentrations of lactic acid in human sweat. To the best of our knowledge, this is the first ever reported example of highly selective nonenzymatic amperometric detection of lactate. Sensitivity, detection limit, and selectivity were characterized, and a nonenzymatic amperometric sensor for selective detection of lactic acid in human sweat was finally demonstrated on a commercial flexible printed electrode.

## 2. Materials and Methods

### 2.1. Surface Modificaiton of Multi-Wall Carbon Nanotube (MWCNT)

The surface of MWCNT was chemically modified to remove the impurities, such as amorphous carbon and graphite particles, and to form the carboxylated surface. The MWCNTs used in this study were purchased at Hanwha Nanotech (CM-95, Incheon, Republic of Korea), with the diameters and lengths ranging 10–15 nm and 10~20 μm, respectively. It was chemically treated with a mixture of 5 M nitric acid and 2 M hydrochloric acid at 80 °C for 15 h. Following that, surface-modified MWCNTs were filtered and washed with distilled water until the pH of filtrate was neutral. Finally, the MWCNTs were dried under vacuum at 70 °C.

### 2.2. Synthesis of MWCNT-Polypyrrole Core-Shell Nanowires

Polypyrrole was uniformly coated on the surface of MWCNTs using the chemical oxidation polymerization method, as reported in previous studies [12,13]. The oxidizing agent, ferric chloride (FeCl_3_, 28.8 mM), was dissolved in 0.1 M hydrochloric acid (100 mL). The pyrrole monomer (16.8 mM) was added to 263 mg of MWCNT and mixed, and then the mixture containing the oxidizing agent was poured onto it. The weight ratio of polypyrrole-to-MWCNT was about 30/70. The mixture was stirred for 3 h at room temperature. Afterwards, it was repeatedly filtered and washed with de-ionized water, ethanol, and acetone several times and then dried at 80 °C under vacuum for 24 h.

### 2.3. Characterization and Electrochemical Measurements

Scanning electron microscopy (SEM) and transmission electron microscopy (TEM) images were examined by a Lectropol-5 scanning electron microscope (FEI, USA) and a TECNAI 20F transmission electron microscope (FEI, USA), respectively. Raman spectra of MWCNT-polypyrrole nanowire were measured using a QE pro-Raman spectrometer (Ocean optics, USA) with a excitation wavelength of 785 nm.

All electrochemical analyses were measured in the potentiostat (Wonatech, Republic of Korea). The cyclic voltammetry (CV) and chronoamperometry (CA) experiments were performed on a typical three-electrode system in a 0.1 M Na_2_SO_4_ aqueous solution purged with N_2_. The working electrode was prepared as follows. The active material and polytetrafluoroethylene (PTFE) were mixed in a weight ratio of 9/1 and the mixture was loaded on a titanium mesh with 10 mg cm^−2^. A platinum wire and Ag/AgCl electrode were used as counter and reference electrodes, respectively. The potential values in this paper were reported against the Ag/AgCl, unless otherwise stated. Before the electrochemical measurements, MWCNT-polypyrrole was activated by applying cyclic potential at a rate of 100 mV s^−1^ in the range of −1.0~1.0 V. CV curves were then recorded between −1 and 1 V at a scan rate of 5 mV s^−1^. To obtain the sensitivity and selectivity to lactic acid, the CA experiment was performed at 0.68 V, and this constant voltage was applied for 1 h before measurement to stabilize the signal.

## 3. Results and Discussion

**Figure 2:**
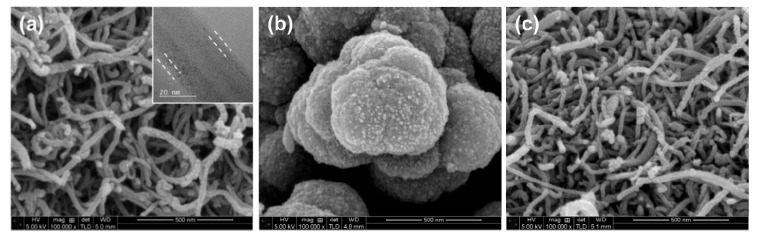
(**a**–**c**) Scanning electron microscopy (SEM) images of (**a**) multi-wall carbon nanotube (MWCNT)-polypyrrole core-shell nanowire, (**b**) polypyrrole, and (**c**) surface-modified MWCNT. The inset of (**a**) is a transmission electron microscopy (TEM) image of corresponding core-shell nanowire.

The morphologies of core-shell nanowires were characterized via scanning electron microscopy (SEM) and transmission electron microscopy (TEM), as shown in Figure 2a–c. Traditionally, MWCNTs have suffered from significant aggregation caused by van der Waals interactions between individual nanotubes, resulting in separated growth of polypyrrole [12,14]. To avoid this problem, the surfaces of the MWCNTs were modified to promote interfacial interactions between pyrrole molecules and the MWCNTs without any addition of surfactants, as shown in Figure S1 [15]. The synthesized MWCNT-polypyrrole compound had an entangled nanowire structure with a uniform diameter and no aggregations or uneven polypyrrole particle distributions (Figure 2a). In the case of polypyrrole without MWCNT, the morphology was shapeless and quite far from the nanowire structure (Figure 2b). The TEM images revealed a coaxial core-shell structure, in which the inner crystalline core is apparently encapsulated by a uniform amorphous shell. The MWCNT core with a crystalline lattice and an amorphous polypyrrole shell are clearly visible. It was also determined that the thickness of the polypyrrole polymerized on the MWCNTs was approximately 4.7 nm by calculating the difference in diameter between the MWCNTs (Figure 2c) and MWCNT-polypyrrole nanowire. These data clearly suggest that the pyrrole monomer was homogeneously polymerized onto the surfaces of MWCNTs to form a core-shell nanowire structure.

**Figure 3:**
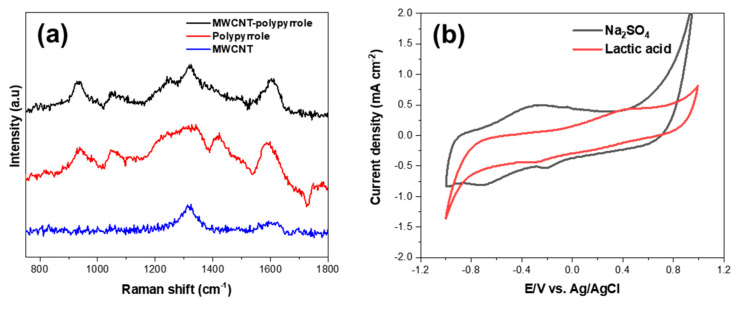
(**a**) Raman spectra of MWCNT-polypyrrole core-shell nanowire, polypyrrole, and surface-modified MWCNT. (**b**) Cyclic voltammetry (CV) curves of MWCNT-polypyrrole core-shell nanowire in Na_2_SO_4_ and lactic acid electrolytes.

Raman spectra were measured for the surface-modified MWCNTs, pure polypyrrole, and MWCNT-polypyrrole core-shell nanowire to confirm the polymerization of pyrrole, as shown in Figure 3a. The surface-modified MWCNTs exhibited two typical peaks attributed to the D and G bands at 1318 and 1603 cm^−1^, respectively, corresponding to disordered sp^2^ microdomains and the symmetric E2g vibrational mode in graphitic structures, respectively [16]. Additionally, the D’ band at 1617 cm^−1^ indicated an increase in defects along the tube body as a result of the surface-modification of MWCNTs [14,17]. Following polymerization of pyrrole on the surface-modified MWCNTs, prominent characteristic Raman bands emerged at 1421, 1164, 1051, 987, and 925 cm^−1^, which were identical to those of pure polypyrrole. The first four bands are attributed to antisymmetrical C-N stretching, N-H in-plane deformation, N-H ring stretching, and symmetrical C-H in-plane bending of polypyrrole, respectively [18,19,20]. The peaks at 925 and 987 cm^−1^ are associated with dications (bipolarons) and radical cations (polarons), respectively [21]. Additionally, the intensity of the bipolarons and polarons increased as additional polypyrrole was coated onto the MWCNT surfaces, resulting in enhanced electrical conductivity (Figure S2). These results confirm the presence of both polypyrrole and surface-modified MWCNTs in the MWCNT-polypyrrole core-shell nanowire.

Figure 3b presents the CV results for the MWCNT-polypyrrole core-shell nanowire. In the 0.1 M Na_2_SO_4_ electrolyte, rounded oxidation and reduction peaks could be observed at −0.28 and −0.73 V. Because polypyrrole is a p-type conductive polymer, only anions are incorporated into the pyrrole backbone to balance the positive charge [22]. Therefore, the oxidative peak resulted from sulfate ion (SO_4_^2−^) doping into the polypyrrole on the MWCNTs and the reduction peak indicated release (dedoping) of the sulfate ions from the polypyrrole. A small reduction peak was also observed at −0.18 V. This peak disappeared when the scan range of CV narrowed from 1.2 V to 0.2 V (Figure S3). Therefore, it should be attributed to the oxidation of polypyrrole at a higher potential.
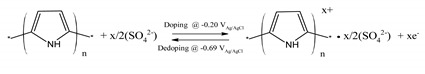
(1)

The lactic acid electrolyte was also applied to CV study. The concentration of lactic acid was increased to overcome the difference of ionization degree between Na_2_SO_4_ and lactic acid. The ionization constant of lactic acid was as low as 1.38 × 10^−4^, while Na_2_SO_4_ was highly ionic and almost completely ionized in water. When utilizing a 1 M lactic acid electrolyte, broad oxidation and reduction peaks could be observed at 0.40 V and −0.30 V, respectively, which are attributed to the doping and dedoping of lactate anions into and from the polypyrrole on the MWCNTs. The doping of lactate ions occurred at a higher potential compared to that of sulfate ions, whereas the dedoping potentials of both ions were similar. Consequently, applying the doping potential of lactate anions was expected to yield a selective current response for lactic acid. 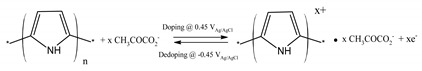
(2)

**Figure 4:**
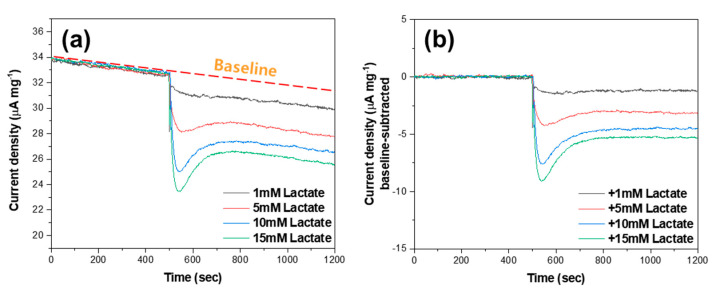
(**a**) Raw chronoamperogram and (**b**) baseline-subtracted chronoamperogram following the addition of lactic acid with the concentration of 1, 5, 10, and 15 mM at a detection potential of 0.68 V.

**Figure 5:**
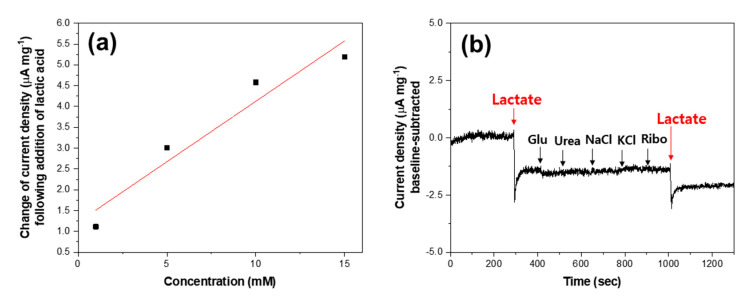
(**a**) Linear relationship between the change of current density and the concentration of lactic acid. (**b**) Amperometric responses to successive additions of 1 mM lactic acid and interfering metabolites of glucose (Glu, 1 mM), urea (1 mM), NaCl (1 mM), KCl (1 mM), and riboflavin (Ribo, 1 mM) at a detection potential of 0.68 V.

Figure 4a presents the chronoamperogram of the MWCNT-polypyrrole core-shell nanowire in the presence of lactic acid at a voltage of 0.68 V. The working voltage of 0.68 V was obtained from a chronoamperometric study at various potentials to achieve a good signal-to-noise ratio (Figure S4). The addition of 1, 5, 10, and 15 mM of lactic acid into a 0.1 M Na_2_SO_4_ electrolyte induced drastic changes in current density from 32.50 mA cm^−2^ to 30.52, 28.49, 27.11, and 26.43 mA cm^−2^, respectively, with overshooting behavior. The MWCNT-polypyrrole core-shell nanowire successfully responded to lactic acid in the millimole range by decreasing the current density at a fixed potential. In order to quantify the current change, the front portions of the curves prior to adding lactic acid were fitted to a third-order polynomial equation. The fitting lines were extrapolated beyond the drastic changes in current density and subtracted from the entire curve to derive baseline-corrected curves, as shown in Figure 4b. In this manner, the differences in current density before and after the addition of lactic acid can be determined accurately. The current density increased monotonically, with an increase in the amount of lactic acid, which means that the current density is concentration dependent. By applying the linear square method, it was determined that the change in current density was linearly proportional to the concentration of lactic acid with a Pearson’s correlation coefficient of 0.97 (Figure 5a). The sensitivity, which was obtained by multiplying the slope with the electrode mass per unit area (10 mg cm^−2^), was determined to be 2.9 μA mM^−1^ cm^−2^. Based on the responses at three times of the standard deviation of blank current density, the limit of detection (LOD) was calculated to be 51 μM. This clearly indicates that the MWCNT-polypyrrole core-shell nanowire is highly suitable for utilization as a quantitative sensor for the detection of lactic acid in human sweat.

The main problem with nonenzymatic electrocatalytic sensors is the simultaneous oxidation of other metabolites coexisting with lactic acid in sweat, such as sodium chloride, potassium chloride, glucose, urea, and riboflavin [23]. When commercial copper oxide (CuO, Aldrich, St. Louis, MO, USA), which is a representative nonenzymatic electrocatalyst, is utilized as a working electrode, the interference effects of these coexisting metabolites on the amperometric response to 1.0 mM of lactic acid is presented in Figure S5. One can see that glucose, urea, and riboflavin significantly affect the current response based on the non-selective oxidation of these metabolites. However, in the case of the MWCNT-polypyrrole core-shell nanowire, lactic acid exhibits a reproducibly distinct current change, while the interfering metabolites generate a negligible current response (Figure 5b). Because neutral or cationic components cannot be incorporated into p-type conducting polymers, the polypyrrole is inert to the interfering species, as shown in Figure 1. As a result, the MWCNT-polypyrrole core-shell nanowire is highly specific to lactic acid, even in the presence of several interfering metabolites found in human sweat. Table 1 lists enzymatic and nonenzymatic electrochemical sensors for the detection of lactic acid reported in recent studies [24,25,26,27,28,29,30,31]. In terms of sensitivity and the LOD, the sensing performance of the MWCNT-polypyrrole core-shell nanowire is comparable to those of enzymatic electrodes with LOx or LDH [24,25,26,27,28,29,30].

Compared to other nonenzymatic sensors, our polypyrrole-MWCNT composite exhibits lower detection of limit for lactic acid and wider range of detection concentration [6] and is demonstrated to apply for human sweat [31].

**Figure 6:**
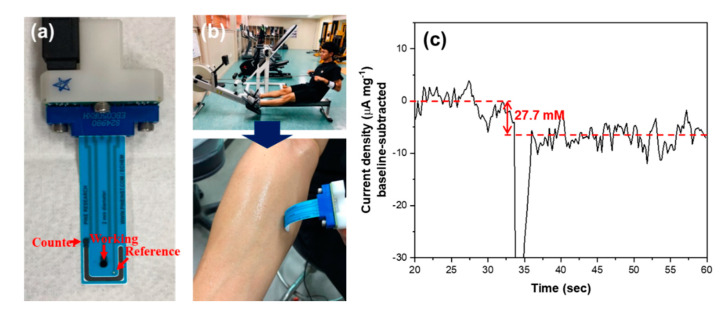
Demonstration of the lactic acid detection from human sweat: (**a**) The flexible printed electrode on which MWCNT-polypyrrole nanowire was deposited; (**b**) collection of human sweat from the volunteer’s forearm after exercise; and (**c**) current response from the collected human sweat.

In order to evaluate the applicability of the MWCNT-polypyrrole core-shell nanowire in the nonenzymatic amperometric sensing of lactic acid in human sweat, the MWCNT-polypyrrole core-shell nanowire was applied to a commercial flexible printed electrode, acquired from PINE research (Figure 6a). Before the test, the electrode was slightly wetted with electrolyte to promote efficient absorption of sweat. When the electrode was in contact with a volunteer’s forearm following exercise, a current response could be observed, as shown in Figure 6b,c. The differences in current density could indicate the concentration of lactic acid (27.7 mM) in a reasonable range. These features of the MWCNT-polypyrrole core-shell nanowire make it a promising material for selective amperometric biosensors for monitoring the concentration of lactic acid in human sweat. The stability and reproducibility of our electrode were assessed with a successive addition of lactic acid, 1 mM. (Figure S6) Although the current response of MWCNT-polypyrrole slightly decreased with the concentration of lactic acid, due to the saturation of lactate doping into the polypyrrole, the current change was still in the linear range of our previous measurement (Figure 4). Furthermore, the current density was not significantly changed under consistent bias, which indicates good stability of our electrode.

## 4. Conclusions

We proposed a nonenzymatic electrochemical detection method for lactic acid in human sweat, utilizing an MWCNT-polypyrrole core-shell nanowire. Polypyrrole was uniformly coated onto the surfaces of MWCNTs with a thickness of 4.7 nm via surface modification and chemical oxidation polymerization. In CV results, the doping of lactate ions into the polypyrrole was observed at approximately 0.45 V, while the doping of sulfate ions was observed at approximately −0.2 V. A chronoamperometric study at 0.68 V in a 0.1 M Na_2_SO_4_ electrolyte revealed good sensing performance for lactic acid with a high sensitivity of 2.9 μA mM^−1^ cm^−2^ and detection limit of 51 μM. Furthermore, the nanowire exhibited excellent selectivity to lactic acid over interfering species, such as sodium chloride, potassium chloride, glucose, urea, and riboflavin, which coexist with lactic acid in sweat. Because polypyrrole is a p-type conducting polymer, into which anions are exclusively doped, leading to charge transfer, it offers selective detection of lactate anions at specific potentials while being inert to the neutral and cationic species contained in human sweat. Finally, a nonenzymatic amperometric sensor for the selective detection of lactic acid in human sweat was implemented by applying the MWCNT-polypyrrole core-shell nanowire to a commercial flexible printed electrode. Using various form-factors and a state-of-art low cost manufacturing technology, our design of electrochemical electrodes will be further developed close to the commercialization of wearable biosensors in the near future. Our experimental results could open the door to novel approaches for the nonenzymatic amperometric detection of lactic acid through the utilization of MWCNT-polypyrrole core-shell nanowires to achieve high selectivity and sensitivity. Moreover, the electrocatlytic property of MWCNT-polypyrrole will be widely used for various applications, such as electrochemical sensors and biofuel power generators.

## Figures and Tables

**Table 1:** Comparison of analytic parameters of various lactate biosensors.

Electrode	Sensitivity (μA/mM)	Limit of Detection (LOD, μM)	Applied Potential (V vs. Ag/AgCl)	Reference
LDH NPs-Au	3.45	0.01	0.10	[24]
LDH-PhNHOH/rGO	10.57	2.50	0.04	[25]
LOx-Pt NPs/CNF/PDDA	36	11.1	0.50	[26]
LOx-CS/MWCNT	3.417	22.6	0.20	[27]
LOx-BSA/GA/Au	37.1	5.0	0.75	[28]
LOx-rGO/DHS	0.0735	2.9	0.10	[29]
LOx-MoS_2_	6.22	17.0	0.30	[30]
3-aminophenylboronic acid (3-APBA)	-	1500	-	[6]
NiO	9.08/cm^2^	53	0.45	[31]
Polypyrrole/MWCNT	2.9	51	0.68	This study

Lactate dehydrogenase nanoparticles (LDH NPs), lactate dehydrogenase (LDH), p-Nitrophenyl moiety (PhNHOH), reduced graphene oxide (rGO), lactate oxidase (LOx), platinum nanoparticles (Pt NPs), carbon nanofiber (CNF), Poly(diallldimethylammonium) chloride (PDDA), Chitosan (CS), multi-walled carbon nanotube (MWCNT), Bovin serum albumin (BSA), glutaraldehyde (GA), N,N’-bis(3,4-dihydroxybenzylidene)-1,2-diaminobenzen (DHS).

## Supplementary Materials

The following are available online at https://www.mdpi.com/2079-6374/10/9/111/s1, Figure S1: Schematic description of the synthesis of MWCNT-polypyrrole core-shell nanowire; Figure S2: Raman spectra of a MWCNT and MWCNT-polypyrrole nanowire with different concentrations of polypyrrole; Figure S3: CV curves of MWCNT-polypyrrole nanowire, polypyrrole and MWCNT in the range from -0.8 V to 0.2 V; Figure S4: Chronoamperograms of MWCNT-polypyrrole nanowire, following the addition of 10 mM lactic acid at various potentials; Figure S5: Amperometric response of CuO to successive additions of 1 mM lactic acid and interfering metabolites of glucose (Glu, 1 mM), urea (1 mM), NaCl (1 mM), KCl (1 mM), and riboflavin (Ribo, 1 mM) at a detection potential of 0.68 V; Figure S5; Figure S6: Stability and reproducibility test for MWCNT-polypyrrole electrode with the repeated addition of 1 mM-lactic acid.
