# Supplementary material for: Selective Nonenzymatic Amperometric Detection of Lactic Acid in Human Sweat Utilizing a Multi-Walled Carbon Nanotube (MWCNT)-Polypyrrole Core-Shell Nanowire
Source: Biosensors (Basel). 2020 Aug 28;10(9):111. doi: 10.3390/bios10090111 (PMC7559985; doi:10.3390/bios10090111)
Supplement: Supplementary file 1 [file biosensors-10-00111-s001.pdf]

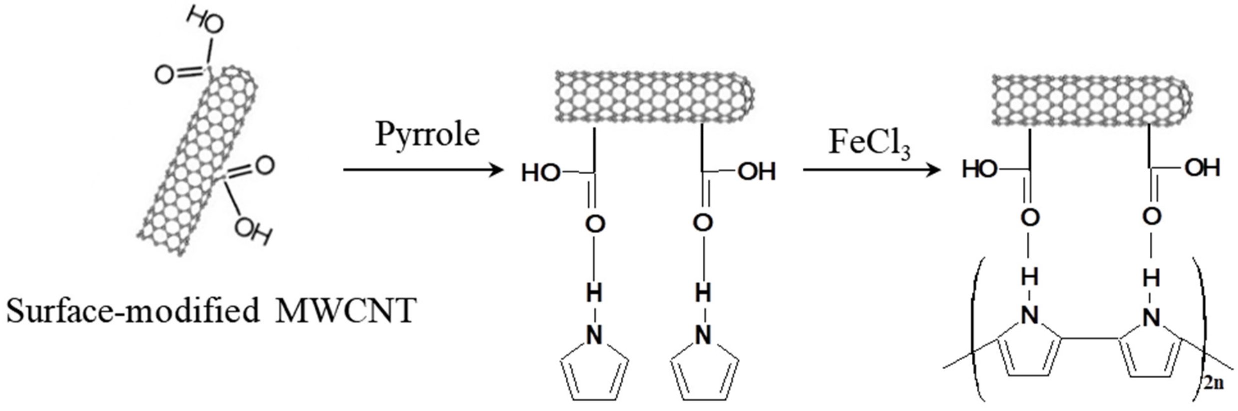

Figure S1. Schematic description of the synthesis of MWCNT-polypyrrole core-shell nanowire.

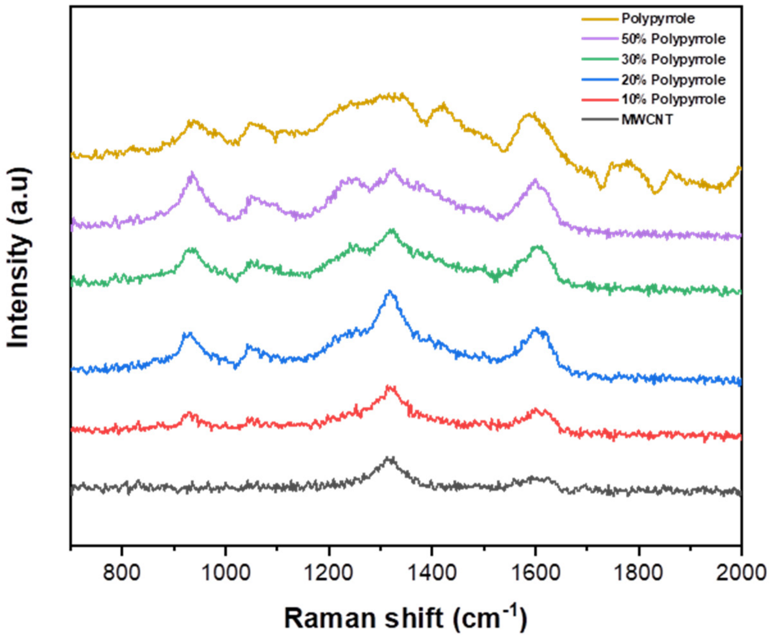

Figure S2. Raman spectra of an MWCNT and MWCNT-polypyrrole nanowire with different concentration of polypyrrole.

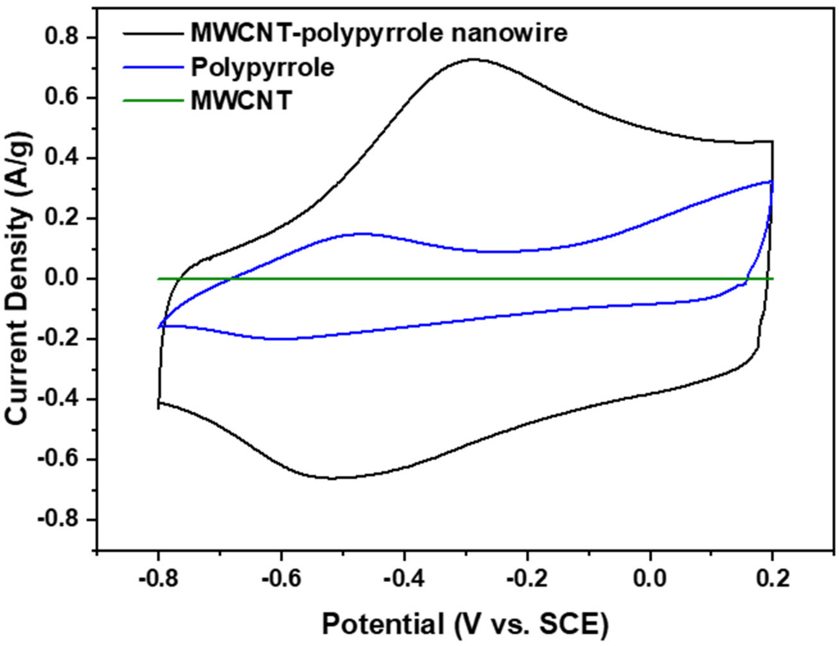

Figure S3. CV curves of MWCNT-polypyrrole nanowire, polypyrrole and MWCNT in the range from -0.8 V to 0.2 V.

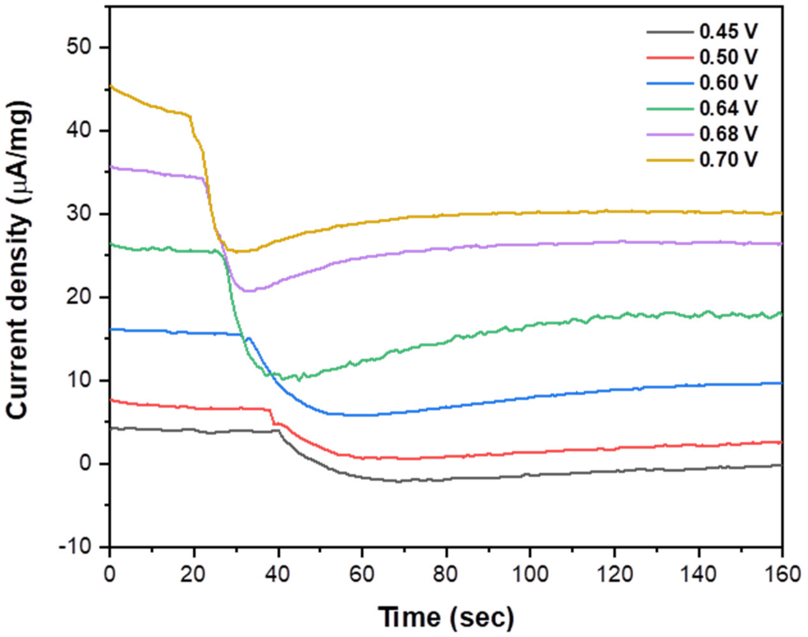

Fig. S4. Chronoamperograms of MWCNT-polypyrrole nanowire following the addition of 10 mM lactic acid at various potential.

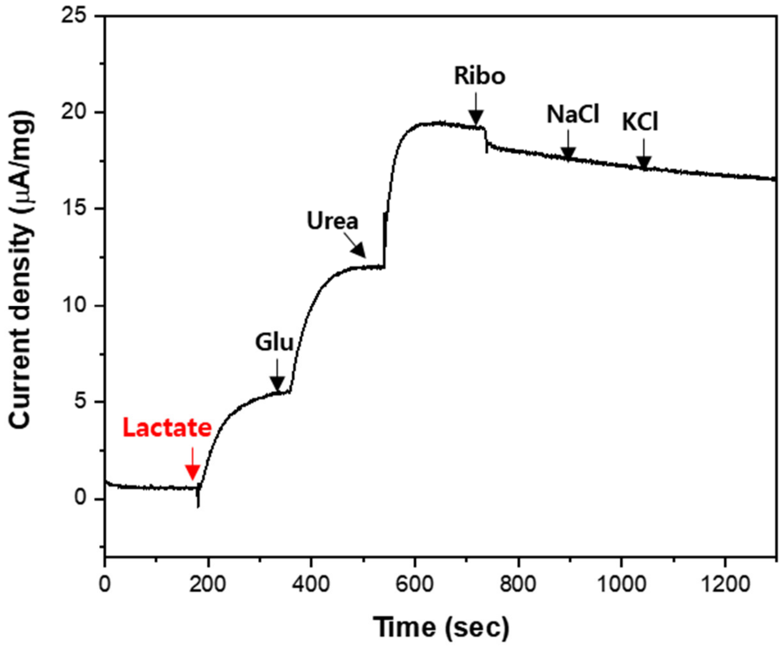

Fig. S5. Amperometric response of CuO to successive additions of 1 mM lactic acid and interfering metabolites of glucose (Glu, 1 mM), urea (1 mM), NaCl (1 mM), KCl (1 mM), and riboflavin (Ribo, 1 mM) at detection potential of 0.68 V.

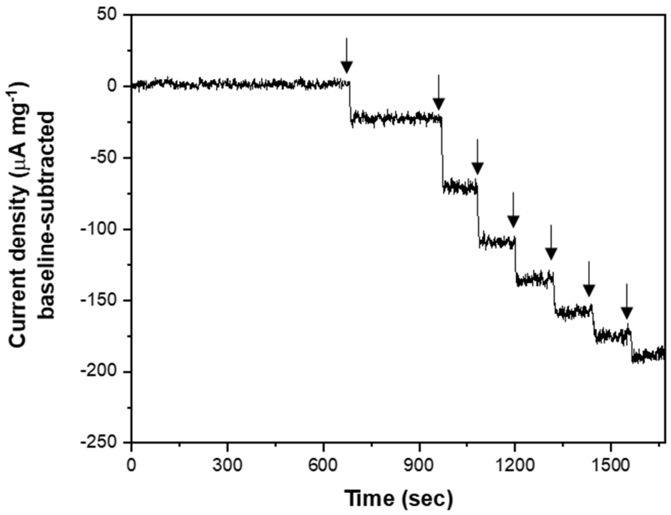

Fig. S6. Stability and reproducibility test for MWCNT-polypyrrole electrode with the repeated addition of 1 mM-lactic acid.
